# ENGAGEMENT OF FAMILY/CARE PARTNERS OF OLDER ADULT LONG-TERM CARE RESIDENTS WITH PHYSIOTHERAPISTS: A SCOPING REVIEW

**DOI:** 10.1093/geroni/igad104.2287

**Published:** 2023-12-21

**Authors:** Denise Connelly, Alexander Stephen, Nicole Guitar

**Affiliations:** Western University, London, Ontario, Canada; University of Western Ontario, London, Ontario, Canada; Western University, London, Ontario, Canada

## Abstract

The aim of this scoping review was to summarize and critically evaluate literature describing the relationship between physical therapists and family/care partners of older adults living in long-term care homes. A librarian-informed search strategy was applied to Medline, EMBASE, Proquest, PsychINFO, SCOPUS and CINAHL databases. Papers were included if they addressed rehabilitation, physical therapists, family/care partners, and long-term care (LTC) setting or equivalent. Five papers were retrieved; quality was mid-to high. Challenges included minimal integration of family/care partners into the long-term care home ’team’, inconsistent resident-centred health goals across staff, and lack of awareness about availability of physical therapy services within long-term care homes. Value for family/care partners as members of the health care team included their historical knowledge of their loved one, ability to perceive and manage behaviour change, signs/symptoms of pain by their loved ones with impaired communication (e.g., dementia diagnosis), and the resource of time and availability to engage in rehabilitation-related care activity. There is little information about partnership with family/care partners of older adults living in long-term care homes. The timing is optimal to explore the engagement of family/care partners in providing care and building rehabilitation capacity within the context of increasing nursing workloads, nurses’ experience of burnout, difficulty in attraction and retention of care staff in long-term care homes, and challenges in funding for and standards in provision of optimal rehabilitation for older adult residents of long-term care homes.

